# High-sensitivity strain sensor based on in-fiber rectangular air bubble

**DOI:** 10.1038/srep07624

**Published:** 2015-01-05

**Authors:** Shen Liu, Kaiming Yang, Yiping Wang, Junle Qu, Changrui Liao, Jun He, Zhengyong Li, Guolu Yin, Bing Sun, Jiangtao Zhou, Guanjun Wang, Jian Tang, Jing Zhao

**Affiliations:** 1 Key Laboratory of Optoelectronic Devices and Systems of Ministry of Education and Guangdong Province, College of Optoelectronic Engineering, Shenzhen University, Shenzhen 518060, China

## Abstract

We demonstrated a unique rectangular air bubble by means of splicing two sections of standard single mode fibers together and tapering the splicing joint. Such an air bubble can be used to develop a promising high-sensitivity strain sensor based on Fabry-Perot interference. The sensitivity of the strain sensor with a cavity length of about 61 μm and a wall thickness of about 1 μm was measured to be up to 43.0 pm/με and is the highest strain sensitivity among the in-fiber FPI-based strain sensors with air cavities reported so far. Moreover, our strain sensor has a very low temperature sensitivity of about 2.0 pm/°C. Thus, the temperature-induced strain measurement error is less than 0.046 με/°C.

Highly sensitive strain measurements by use of optical fiber sensors are very attractive in many applications such as structure health monitoring, aerospace, and nanotechnology^1^,^2^,^3^. Such a measurement can be realized by use of fiber-device-based schemes, e.g. fiber in-line interferometers, fiber Bragg gratings (FBGs), and long period fiber gratings (LPFGs). The measured strain sensitivity was typically less than 7.0 pm/με for a fiber Mach-Zehnder interferometer^4^,^5^, about 2.0 pm/με for an FBG^6^,^7^, and about 10 pm/με for an LPFG^8^,^9^,^10^,^11^. In addition, the cross-sensitivity between strain and temperature was hardly overcome in these sensor schemes. Recently, Fabry-Perot interferometers (FPIs) based on an in-fiber air cavity were proved to be outstanding in lots of sensing applications, such as strain measurements^12^,^13^,^14^,^15^,^16^,^17^,^18^,^19^, refractive index (RI) measurements^20^,^21^ and pressure measurements^22^,^23^, due to the advantages of simple configuration, high sensitivity, compact size, and low temperature cross-sensitivity. However, it is very difficult to directly create an air cavity in the fiber. A few complex methods have been proposed to create in-fiber air cavities by use of silica hollow tube^18^ or photonic crystal fibers (PCFs)^14^,^17^,^21^, direct micromachining using focused femtosecond laser beam^5^,^13^,^20^, and assisted chemical etching^24^. The above-mentioned FPIs usually require special optical fibers, high-cost micromachining equipment, or/and hazardous acid corrosion treatment. Recently, we reported a novel and simple technique for fabricating air-cavity-based FPIs that can be used to develop a strain sensor with a high sensitivity of up to 6.0 pm/με^19^. The air cavity was created by use of a common commercial fusion splicer to splice together two sections of standard single mode fibers (SMFs) with easy pretreatment on the fiber ends.

In this paper, we demonstrated an improved technique to create a unique rectangular air bubble by means of splicing together two sections of SMF tapers. The rectangular air-bubble-based FPI with a cavity length of about 61 μm exhibits a high strain sensitivity of 43.0 pm/με around the wavelength of 1550 nm. Moreover, such a strain sensor has a very low temperature sensitivity of about 2.0 pm/°C, where a lower temperature cross-sensitivity of 0.046 με/°C than that reported in ref. 19 was obtained. So the rectangular air-bubble-based FPI could be used to develop a promising high-sensitivity strain sensor with a low temperature sensitivity.

## Experiments

**Figure 1:**
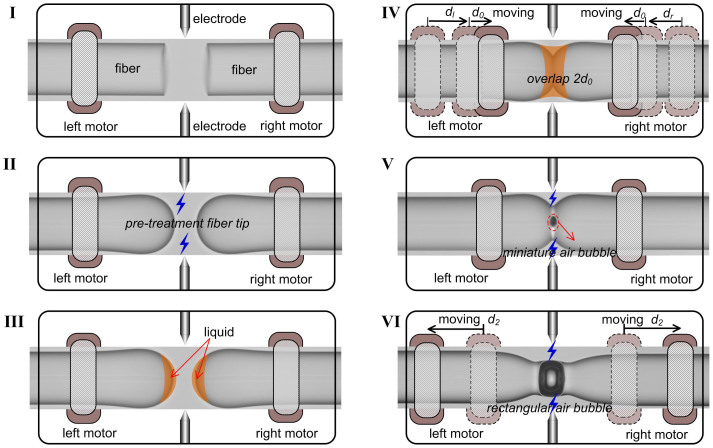
Schematic diagrams of fabrication process of in-fiber FPI based on an air bubble.

Fig. 1 illustrates the fabrication process of our proposed rectangular air bubble in a SMF, which involves six steps. In step 1, as shown in Fig. 1(I), two sections of SMFs (Corning SMF-28) with cleaved ends were placed in the left and right fiber holds of a commercial fusion splicer (Fujikura FSM-60S). In step 2, as shown in Fig. 1(II), the two fiber ends were reshaped into hemispherical and smooth ends via electrical arc discharge in order to enlarge the surface area of the fiber ends for coating sufficient liquid on the fiber ends. In step 3, as shown in Fig. 1(III), the two hemispherical fiber ends were immersed into a commercial refractive index matching liquid (Cargille Labs, http://www.cargille.com) to coat a liquid film on the end surface. The two sections of SMFs with liquid-coated ends were again placed in the left and right fiber holders of the fusion splicer. In step 4, as shown in Fig. 1(IV), the left and right fiber ends were moved toward each other until an overlap of 2d_0_ was achieved at the touching region of the two fiber ends by means of carefully controlling the movement of the left and right motors of the fusion splicer, where d_l_ and d_r_ indicate the moved distance of the left and right fiber ends, respectively, before the two fiber ends touched, and d_0_ indicates the moved distance of each fiber end after the two fiber ends touched. It should be noted that the value of “d_0_” here is less than that of “d” reported in Ref. 19, which means a smaller axial stress was applied to the fiber ends at the touching region. In step 5, the liquid-coated fiber ends were aligned with each other and then spliced together via electrical arc discharge with a fusion current of 18 mA and a fusion time of 1000 ms. Consequently, as shown in Fig. 1(V), a miniature air bubble with a diameter of about 10 μm was formed in the spliced region with an abrupt taper shape, resulting from the evaporation of the liquid coated on the fiber end faces and the fusion of silica during arc discharge. In step 6, the left and right fiber holders were moved backward to each other with a distance of d_2_ in order to apply an axial tensile stress to the air bubble region located in the middle of fused taper, as shown in Fig. 1(VI). And then, an electrical arc discharge with a fusion current of 18 mA and a fusion time of 750 ms was implemented at the middle of air bubble. During the arc discharge, the silica wall of the air bubble melted due to the arc-discharge-induced high temperature and the air in the bubble thermally expanded, resulting in a rectangular air bubble due to the pre-applied axial tensile stress, as shown in Fig. 1(VI). And the thickness of the silica wall was gradually decreased by means of repeating the step 6. Using the method above, we have successfully fabricated a few in-fiber rectangular air bubble samples. In the whole fabrication process, only a common fusion splicer was employed, and no additional device was required.

**Figure 2:**
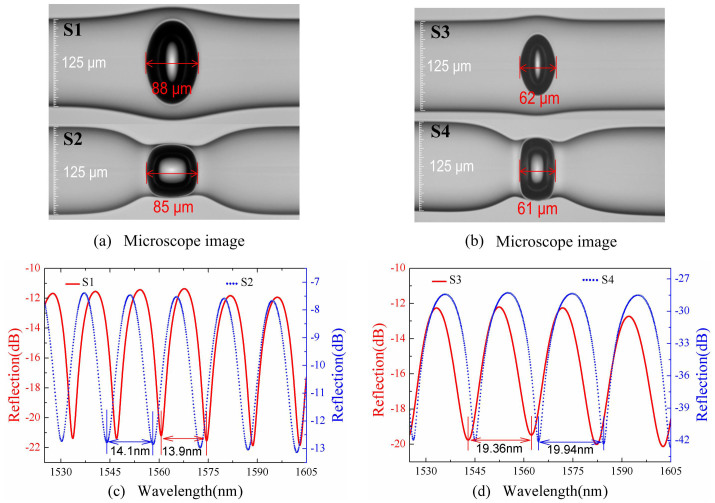
Four in-fiber air bubble samples. (a) and (b) Microscope images of the created elliptical air bubbles, i.e. S1 and S3, and the created rectangular air bubbles, i.e. S2 and S4, with a cavity length of about 88, 62, 85 and 61 μm, respectively; (c) and (d) The corresponding reflection spectra of the air-cavity-based FPI samples.

To investigate the strain sensitivity difference between the rectangular and elliptical air bubbles, as shown in Fig. 2, two rectangular air bubble sample, i.e. S2 and S4, were created by the technique above, and another two elliptical air bubbles, i.e. S1 and S3, were created by the technique reported previously in ref. 19. Each pair of the rectangular and elliptical air bubbles, e.g. S1 and S2, or S3 and S4, has an approximate cavity length of about 88 μm (85 μm) or 62 μm (61 μm) to maintain similar measured parameters. The wall thickness of S1, S2, S3 and S4 was measured to be about 23, 1, 28, and 1 μm, respectively. A broadband light source, a 3-dB fiber coupler, and an optical spectrum analyzer (YOKOGAWA AQ6370C) with a resolution of 0.01 nm were employed to measure the reflection spectra of these two pairs of air bubbles. As shown in Figs. 2(c) and 2(d), clear interference fringes were observed in the reflection spectra of the air bubble samples, S1, S2, S3 and S4. And the corresponding free spectra range (FSR) of the interference fringes around 1550 nm was measured to be 13.9, 14.1, 19.36 and 19.94 nm, respectively. According to the measured FSR above and the equation of *FSR* = *λ*^2^/(2*nL*), the cavity length, *L*, of the four samples were calculated to be 85.2, 86.4, 62.0 and 60.2 μm, respectively, which agrees well with the values measured from Figs. 2(a) and 2(b).

## Results and discussion

**Figure 3:**
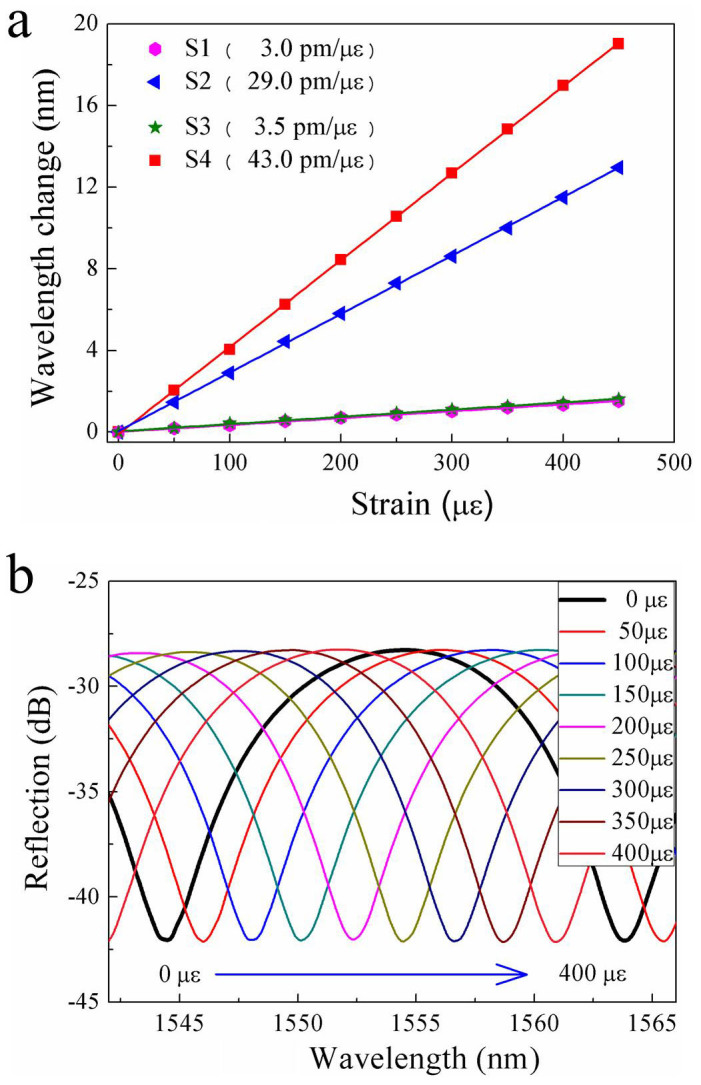
Strain response of the four air bubble samples. (a) Wavelength change of the interference fringe around 1550 nm as a function of tensile strain applied to the air-cavity-based FPI samples: (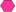) S1, (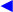) S2, (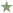) S3 and (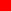) S4, with a cavity length of 88, 85, 62 and 61 μm, respectively;(b) Reflection spectrum evolution of the rectangular air bubble sample, i.e. S4, while the tensile strain increases from 0 to 400 με.

One end of the air bubble sample was fixed, and another end was attached to a translation stage with a resolution of 10 μm. A tensile strain was applied to the air bubble sample by moving the translation stage away from the fixed one at room temperature. The wavelength shift of the fringe dip around 1550 nm was measured while the tensile strain was increased from 0 to 450 με with a step of 50 με. As shown in Fig. 3, the fringe dips of the four samples shifted linearly toward a longer wavelength with the increased tensile strain. The strain sensitivity of S1, S2, S3 and S4 was calculated to be 3.0, 29.0, 3.5 and 43.0 pm/με, respectively, by applying a linear fitting of the experimental data. So, the strain sensitivity of the rectangular air bubble samples, i.e. S2 and S4, is almost nine and twelve times higher than that of the elliptical air bubbles, i.e. S1 and S3, respectively, which indicates that the rectangular air bubble can significantly enhance the strain sensitivity of the air-cavity-based FPI.

**Figure 4:**
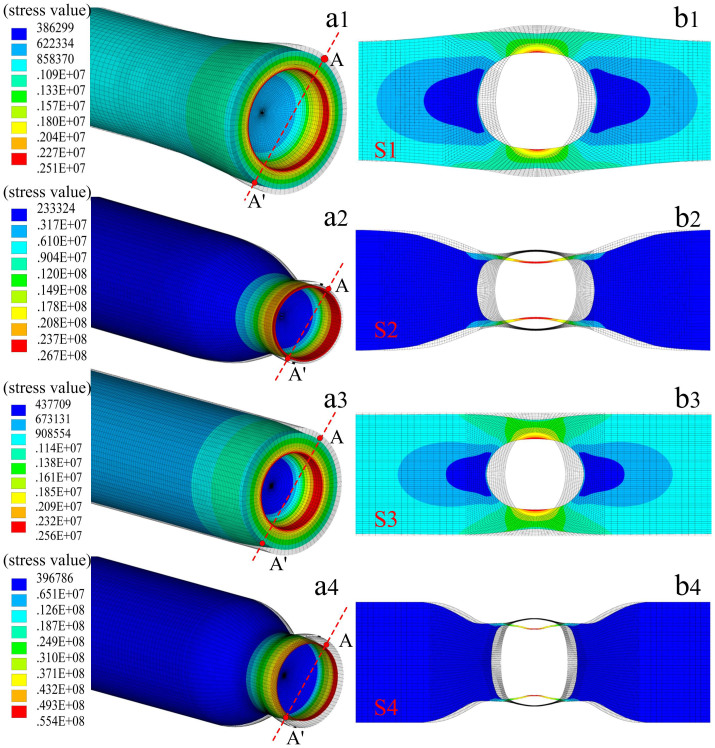
Stress distribution of the four air bubble samples, i.e. S1, S2, S3, and S4, under an applied tensile strain of 1 με. (a1), (a2), (a3) and (a4) Three-dimensional stress contours and (b1), (b2), (b3), and (b4) Two-dimensional stress contours on the A–A′ section plane, where Young's modulus of silica is 73 Gpa, Poisson's ratio is 0.17, silica density is 2700 Kg/m^3^.

**Figure 5:**
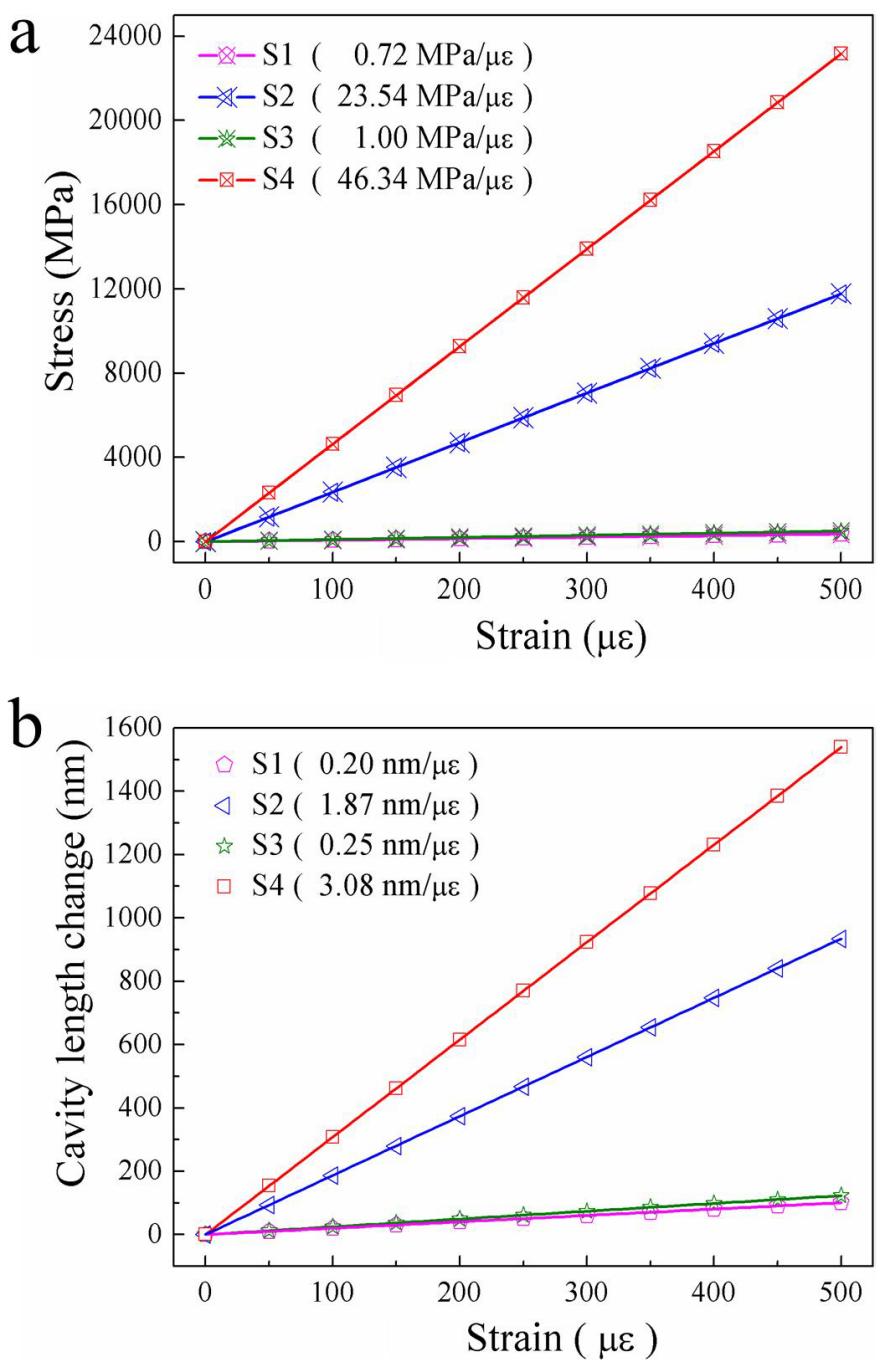
Numerical simulation for the stress concentration and the cavity length change of the four air bubble samples under an applied tensile strain. (a) Calculated stress at the ‘A' point on the outside surface of each air bubble versus the applied strain, (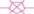) S1, (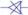) S2, (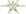) S3, and (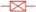) S4; (b) Calculated cavity length change of each air bubble versus the applied strain, (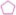) S1, (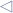) S2, (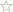) S3, and (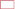)S4.

In order to study the stress distribution and the deformation of the air cavity under an applied tensile strain, simulation models were established by use of a commercial software, i.e. ANSYS, and the measured sizes of the four air bubble samples were illustrated in Figs. 2(a) and 2(b). Fig. 4 illustrate the three- and two dimensional stress contours of the four air bubble samples, i.e. S1, S2, S3, and S4, with a tensile strain of 1 με, which indicates the calculated stress distribution in different parts of each air bubble sample. While the applied tensile strain increases, as shown in Fig. 5(a), the calculated stress at the ‘A' point on the outside surface of the two rectangular air bubbles, i.e. S2 and S4, increases linearly with a slope of 23.54 and 46.34 MPa/με, respectively, in contrast, that of the two elliptical air bubbles, i.e. S1 and S3, hardly increased with a very low slope of 0.72 and 1.00 MPa/με, respectively. So the stress distributed on the silica wall of an in-fiber air bubble sharply increases with the applied tensile strain in case such an air bubble has a rectangular shape and is created in the fused taper. Moreover, while the applied tensile strain increases, as shown in Fig. 5(b), the calculated cavity of the rectangular air bubble samples, i.e. S2 and S4, sharply lengthened with a slope of 1.87, and 3.08 nm/με, respectively, in contrast, that of the elliptical air bubble samples, i.e. S1 and S3, slowly lengthened with a low slope of 0.20 and 0.25 nm/με, respectively. So the calculated cavity length sensitivity, i.e. strain sensitivity, of the rectangular air bubble samples, i.e. S2 and S4, is nine and twelve times higher than that of the elliptical air bubble samples, i.e. S1 and S3, respectively, which agrees well with the measured strain sensitivities, as shown in Fig. 3(a).

**Figure 6:**
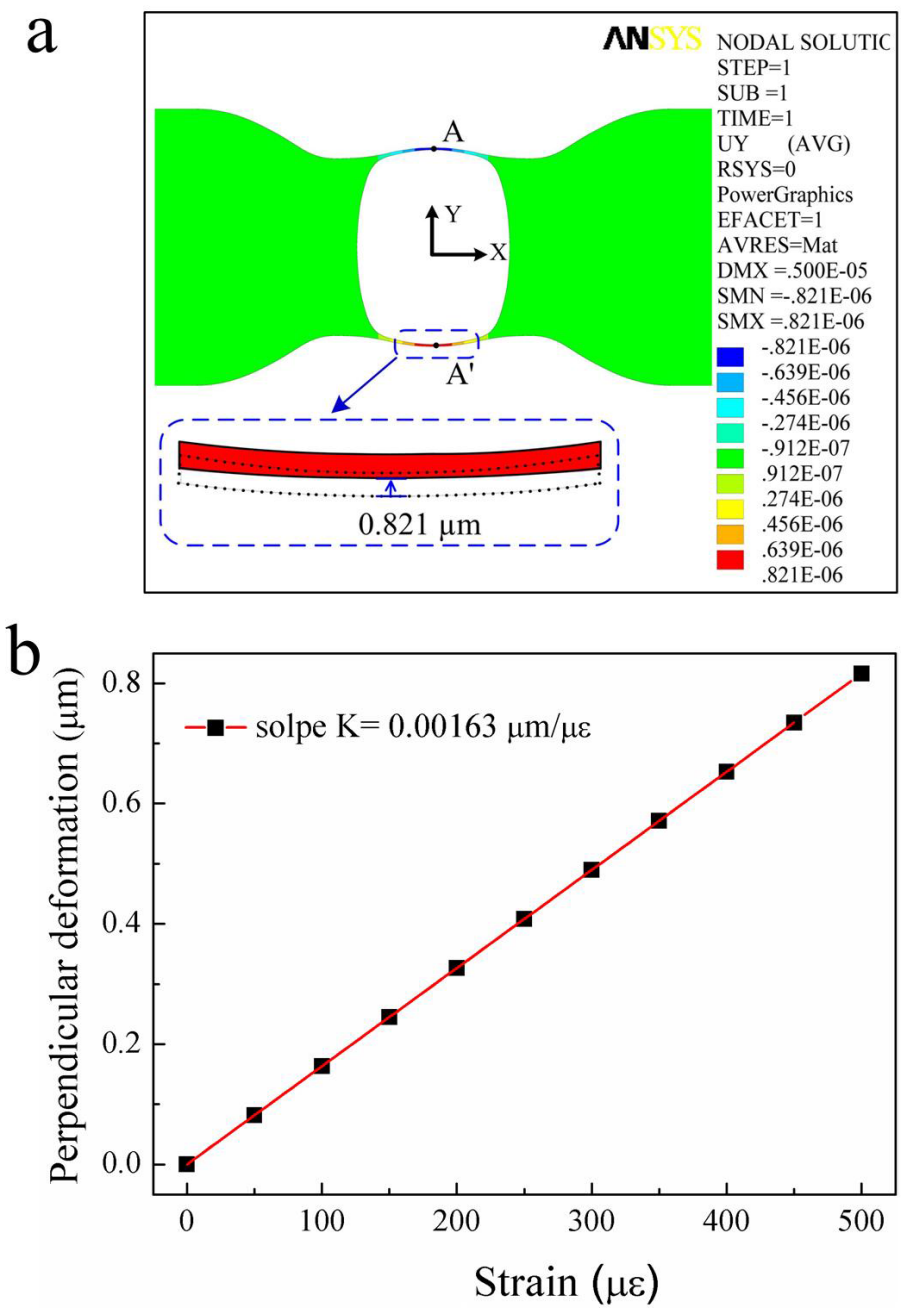
Numerical simulation for the perpendicular deformation of the sample (S4). (a) Simulated perpendicular deformation on the A–A′ section plane, illustrated in Fig. 4 (a4), of the sample (S4) with a tensile strain of 500 με, where the maximum perpendicular deformation is 0.821 μm; (b) Calculated perpendicular deformation of the sample (S4) versus an applied tensile strain from 0 to 500 με.

It was very difficult to measure the practical perpendicular deformation of the air bubble sample during applying a tensile strain. So we simulated the perpendicular deformation of the rectangular air bubble sample (S4) with a tensile strain. The perpendicular deformation contours on the cross-section of S4 with a tensile strain of 500 με is shown in Fig. 6 (a), where the dotted and solid line in the inset illustrates the outline of the air bubble wall before and after the tensile strain was applied, respectively. It can be seen from the inset of Fig. 6(a) that a maximum perpendicular deformation of 0.821 μm occurred at the A (A') point of S4. Moreover, while the applied tensile strain increases from 0 to 500 με, as shown in Fig. 6 (b), the calculated perpendicular deformation at the A (A') point of S4 increased linearly with a slope of 1.63 nm/με.

**Figure 7:**
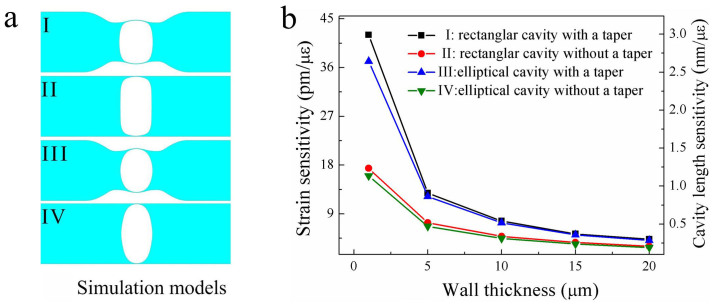
Numerical simulation for different types of in-fiber air bubbles. (a) Four types of air bubble models with a same cavity length of 61 μm, **I**: a rectangular air bubble with a tapered section, **II**: a rectangular air bubble without a tapered section, **III**: an elliptical air bubble with a tapered section, and **IV**: an elliptical air bubble without a tapered section; (b) Strain sensitivity (Left axis), i.e. the wavelength sensitivity of the interference fringes, and cavity length sensitivity (Right axis) of the air bubbles (**I**, **II**, **III** and **IV**) with different wall thickness.

**Figure 8:**
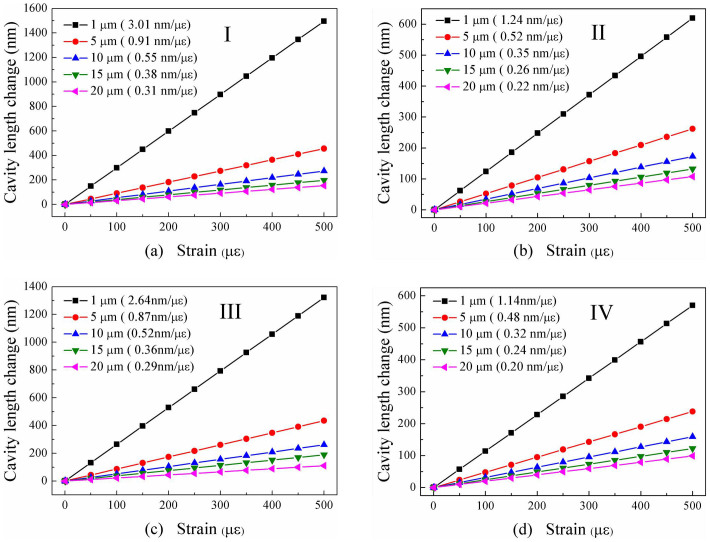
Calculated cavity length change of each air bubble model, i.e. (a) **I**,(b) **II**, (c) **III** and (d) **IV**, with different wall thickness (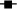) 1 μm, (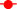) 5 μm, (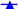) 10 μm, (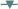) 15 μm and (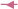) 20 μm while the applied tensile strain increases from 0 to 500 με.

As shown in Fig. 7 (a), four types of in-fiber air bubble models with a same cavity length of 61 μm was designed to investigate quantitatively the dependency of their strain sensitivity on the wall thickness and the cavity shape, where the tapered sections of Model-I and Model-III are with a same size. We calculated the cavity length change of each air bubble model (**I**, **II**, **III** and **IV**) with different wall thickness (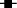) 1 μm, (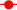) 5 μm, (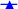) 10 μm, (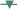) 15 μm and (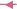) 20 μm while an applied tensile strain increases from 0 to 500 με. As shown in Fig. 8(a), the cavity length sensitivity of the rectangular air bubble with a tapered section (I) and a wall thickness of 1, 5, 10, 15, and 20 μm is 3.01, 0.91, 0.55, 0.38 and 0.31 μm/με, respectively. As shown in Fig. 8(b), the cavity length sensitivity of the rectangular air bubble without a tapered section (**II**) and with a wall thickness of 1, 5, 10, 15, and 20 μm is 1.24, 0.52, 0.35 and 0.26 μm/με, respectively. As shown in Fig. 8(c), the cavity length sensitivity of the elliptical air bubble with a tapered section (**III**) and a wall thickness of 1, 5, 10, 15, and 20 μm is 2.64, 0.87, 0.52, 0.36 and 0.29 μm/με, respectively. As shown in Fig. 8(d), the cavity length sensitivity of the elliptical air bubble without a tapered section (**IV**) and with a wall thickness of 1, 5, 10, 15, and 20 μm is 1.14, 0.48, 0.32, 0.24 and 0.20 μm/με, respectively.

The cavity length sensitivities of each type of air bubble model above versus different wall thickness are summarized in Fig. 7(b), where the corresponding strain sensitivity (left axis), i.e. the wavelength sensitivity of the interference fringes, was calculated by use of Fabry-Perot interference principle, i.e. *dλ/dε* = (*λ_m_*/*L*)*dL*/*dε*, where *λ_m_* is the wavelength of the m^th^ order interference dip^20^. As shown in Fig. 7 (b), (1) for each type of air bubbles, the wall thickness is smaller, the strain sensitivity is higher; (2) both rectangular and elliptical air bubbles with a tapered section exhibits a much higher stain sensitivity than those without a tapered section; (3) the rectangular air bubble with or without a tapered section exhibits a litter higher strain sensitivity than the elliptical air bubble with or without a tapered section, respectively. So the strain sensitivity enhancement of the air bubble owes to the reduction of the wall thickness, rather than the shape of the air cavity. In our experiments, it was to reduce the wall thickness of the air cavity and thus enhance its strain sensitivity that an elliptical air bubble was reshaped into a rectangle air bubble by means of tapering the air cavity section.

A few types of in-fiber FPI-based strain sensors with different air cavities and their sensitivities were presented in Table 1. It can be seen from Table 1 that, to the best of our knowledge, our current strain sensor exhibits the highest sensitivity of 43.0 pm/με, which owes that our proposed sensor is based on a rectangular air bubble with a very thin wall thickness of about 1 μm and a tapered section.

**Figure 9:**
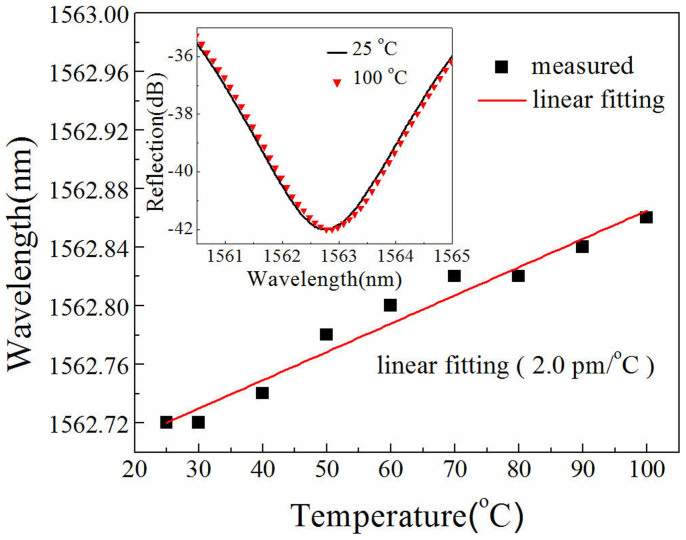
Wavelength shift of the interference dip at the wavelength of about 1560 nm of the sample S4 versus ambient temperature. Inset: Reflection spectra of S4 at 25 and 100°C.

Temperature response of the rectangular air bubble sample, i.e. S4, also was investigated by placing it into an electrical oven and gradually raising temperature from 25 to 100°C with a step of 10°C. As shown in Fig. 9, the dip wavelength of the interference fringe in the reflection spectrum of S4 slowly shifted linearly toward a longer wavelength with a low sensitivity of about 2.0 pm/°C. According to the strain and temperature sensitivity of the interference fringe, the temperature-induced strain measurement error is less than 0.046 με/°C in case no temperature compensation is done.

## Conclusions

A novel technique was demonstrated to fabricate a unique rectangular air bubble with a very thin wall thickness of about 1 μm and a tapered section in an optical fiber. Such a rectangular air bubble can be used to develop a promising high-sensitivity strain sensor based on Fabry-Perot interference. The sensitivity of the strain sensor is up to 43.0 pm/με and is the highest sensitivity among the in-fiber FPI-based strain sensors with air cavities reported so far. Moreover, such a strain sensor has a very low temperature sensitivity of about 2.0 pm/°C, and therefore the cross-sensitivity between strain and temperature was reduced to a very low value of 0.046 με/°C.

## Figures and Tables

**Table 1:** A few types of in-fiber FPI-based strain sensors with air cavities

cavity structure	strain sensitivity	cavity size	reference
elliptical air bubble	6.0 pm/με	46 μm	Ref. 19
hollow-core ring PCF	15.4 pm/με	13 μm	Ref. 17
micro-bubble	4 pm/με	91 μm	Ref. 15
air hole	6 pm/με	80 μm	Ref. 13
spheroidal cavity+PCF	2.7 pm/με	58 μm	Ref. 14
spheroidal cavity	10.3 pm/με	10 μm	Ref. 16
rectangular air bubble	43 pm/με	61 μm	this work
